# Developmental Comparative Analysis of Enteroendocrine Hormone Immunoreactive Cells in the Abomasum and Small Intestine of Holstein–Friesian Bulls

**DOI:** 10.3390/ani16091407

**Published:** 2026-05-04

**Authors:** Ligia Janicka, Aleksandra Dajnowska, Katarzyna Woźniak, Cezary Osiak-Wicha, Katarzyna Kras, Iwona Łuszczewska-Sierakowska, Marcin B. Arciszewski

**Affiliations:** 1 Department of Animal Anatomy and Histology, Faculty of Veterinary Medicine, University of Life Sciences in Lublin, Akademicka 12, 20-950 Lublin, Poland; ligia.janicka@up.edu.pl (L.J.); katarzyna.wozniak@up.edu.pl (K.W.); cezary.wicha@up.edu.pl (C.O.-W.); katarzyna.kras@up.edu.pl (K.K.); mb.arciszewski@wp.pl (M.B.A.); 2 Department of Correct Clinical and Imaging Anatomy, Faculty of Medical Sciences, Medical University of Lublin, 20-090 Lublin, Poland; iwona.luszczewska-sierakowska@umlub.pl

**Keywords:** abomasum, cattle, development, digestive physiology, enteroendocrine cells, gastrointestinal tract, small intestine

## Abstract

Within the complex regulatory network of the gastrointestinal tract, enteroendocrine cells play a fundamental role in coordinating digestive processes and maintaining metabolic balance. These cells produce a wide range of hormones that influence secretion, motility, and nutrient utilization, constituting an essential component of gastrointestinal physiology in ruminants. Despite the growing interest in the role of endocrine regulation in digestive system function, comprehensive studies evaluating enteroendocrine cell populations across different segments of the gastrointestinal tract in cattle remain limited. In particular, the organization of these cell populations in key digestive segments such as the abomasum and small intestine requires further investigation to better understand their functional significance. The aim of the present study was to provide a comparative characterization of selected enteroendocrine cell populations in the abomasum and small intestine of young and adult Holstein–Friesian bulls. Tissue samples collected from the abomasum, duodenum, jejunum, and ileum were subjected to histological and immunohistochemical analyses. Chromogranin A (CgA) was used as a general marker of enteroendocrine cells, and the number of cells immunoreactive to CgA as well as to gastrin, secretin, somatostatin (SOM), glucose-dependent insulinotropic polypeptide (GIP), and glucagon-like peptide-1 (GLP-1) was quantified in the examined segments of the gastrointestinal tract. All types of immunoreactive cells were counted using a standardized quantitative method based on the assessment of positively stained cells in randomly selected microscopic fields of histological sections. The results revealed clear differences in the number of enteroendocrine cells between the analyzed segments of the gastrointestinal tract as well as between the studied age groups. These findings indicate regional and age-related variation in enteroendocrine cell populations within the gastrointestinal tract and highlight their association with the functional organization of individual segments of the digestive system.

## Simple Summary

The gastrointestinal tract plays a key role in the digestion and utilization of nutrients, and its proper functioning is essential for the growth, health, and productivity of animals. In the present study, the number of enteroendocrine cells, including cells immunoreactive to selected hormones, was compared in the abomasum and small intestine of young (7–8 months) and adult (20–24 months) Holstein–Friesian bulls. Clear differences were observed between age groups in the number of these cells in particular segments of the gastrointestinal tract, as well as differences among the individual segments within the group. The results indicate that age is an important factor associated with the functioning of the digestive system. Understanding these relationships may contribute to the development of more precise nutritional strategies and to supporting the health and productivity of cattle.

## 1. Introduction

In ruminants, the primary fermentation processes take place in the forestomachs, whereas the abomasum and small intestine are responsible for enzymatic digestion and the preparation of nutrients for absorption [1]. Within these regions, the gastrointestinal tract is subject to multifaceted hormonal regulation, which influences its motility, secretory activity, and the rate of metabolic processes [2]. The structure and function of the gastrointestinal tract undergo substantial changes during development, which affect feed utilization and overall physiological status [3]. Although digestive mechanisms in calves achieve functional maturity during the first months of life and reach a relatively stable physiological state during later juvenile development [4], the enzymatic profile of the gastrointestinal tract and the composition of the ruminal microbiota remain different from those of adult animals [5]. At this age, hormonal regulation becomes increasingly important in cattle, and a mature network of enteroendocrine cells (EECs) is established [3].

The gastrointestinal tract exhibits marked interspecies differences that reflect distinct digestive strategies. In ruminants, digestion relies primarily on fermentation processes occurring in a complex, multi-chambered stomach, whereas in monogastric animals, such as pigs, rodents, and humans, feed is digested mainly through enzymatic processes in a single-chambered stomach [6]. Consequently, the development of the gastrointestinal endocrine system also differs among species, and in ruminants its maturation occurs more gradually and is closely associated with the functional development of the forestomachs and the establishment of microbial fermentation [7].

EECs are widely distributed throughout the gastrointestinal tract and play a key role in regulating motility, secretion, appetite, and metabolism through hormonal, paracrine, and neuronal signaling [8]. Chromogranin A (CgA), a marker of the entire EEC population, enables the assessment of their abundance and distribution across different regions of the gastrointestinal tract [9]. Gastrin, a hormone secreted by G cells, is primarily localized in the pyloric region of the abomasum, where it stimulates hydrochloric acid secretion and supports mucosal epithelial renewal [10,11]. Secretin, synthesized predominantly in the duodenum and to a lesser extent in the proximal jejunum, is responsible for neutralizing acidic gastric contents and initiates the secretion of bicarbonates and bile [12]. The incretin hormones, glucose-dependent insulinotropic polypeptide (GIP) and glucagon-like peptide-1 (GLP-1), secreted by K and L cells of the small intestine, modulate insulin responses, glucose homeostasis, and intestinal motility, and their significant metabolic role has also been demonstrated in ruminants [13,14]. Somatostatin (SOM), present in the abomasum and small intestine, inhibits the secretion of numerous gastrointestinal hormones, including gastrin and secretin, thereby serving as a regulator of endocrine balance [15].

The distribution of these hormones reflects the functional specialization of different gastrointestinal segments [16]. In the abomasum, regulatory processes are dominated by gastrin and SOM, which play key roles in controlling gastric secretion and function [17]. In the duodenum, mechanisms involved in neutralizing digesta and mediating the early response to nutrients are of particular importance, which justifies the analysis of secretin, incretins, SOM, and CgA [18,19]. In contrast, the jejunum and ileum, which serve as the primary sites of nutrient absorption and metabolic signal integration, are characterized by the presence of cells secreting GIP, GLP-1, SOM, and the marker CgA [20,21].

Despite increasing interest in gastrointestinal endocrinology in ruminants, most previous studies have focused on individual hormones [22] and were conducted under variable environmental and feeding conditions [23]. Studies simultaneously assessing multiple EEC populations across different intestinal segments are limited, and comparative analyses between young and adult animals remain scarce [24,25]. This gap hinders a comprehensive understanding of the development of the gut–endocrine axis and its significance for metabolism, growth, and health in cattle.

The aim of the present study was an age-dependent, comparative assessment of the number and distribution of selected enteroendocrine hormone-immunoreactive cells in the abomasum and in individual segments of the small intestine of Holstein–Friesian bulls. The analysis included the detection of gastrin, SOM, and CgA in the abomasum, as well as secretin, GIP, GLP-1, SOM, and CgA in the duodenum, jejunum, and ileum. By comparing the results obtained in young and adult animals, the study aimed to identify age-related differences and to determine how gastrointestinal maturation influences the organization and distribution of the enteroendocrine system in key regions responsible for digestive regulation in ruminants. It was hypothesized that young bulls, due to the ongoing maturation of the gastrointestinal tract and higher metabolic demands, exhibit differences in the number and distribution of selected enteroendocrine cell populations compared with adult individuals. It was further hypothesized that adult animals display a more stable and regionally differentiated pattern of enteroendocrine cell distribution, reflecting the established function of the mature ruminant. Additionally, age-related differences in the organization of the enteroendocrine system may be associated with differences in forestomach fermentation and metabolic demands between the growth phase and adulthood.

## 2. Materials and Methods

### 2.1. Animals

The study was conducted on Holstein–Friesian male cattle belonging to two age groups: 6 adult individuals aged 20–24 months (768 ± 46 kg) and 6 calves aged 7–8 months (218 ± 23 kg). The use of males allowed the elimination of cyclical hormonal fluctuations typical of females, which could otherwise interfere with the assessment of the enteroendocrine profile. The Holstein–Friesian breed was selected due to its dominant position in national cattle production, making it a key research model for studying ruminant physiology and health.

All animals originated from a single farm where a uniform feeding system and similar environmental conditions were maintained. A semi-intensive feeding model was used, including a grazing period followed by the administration of a full-ration total mixed ration (TMR), in accordance with the description by Włodarczyk et al. (2011) [26]. The animals were kept and fed routinely for this farm, without experimental interventions.

The sample size was determined according to Mead’s heuristic [27,28], which indicated that 6 animals in each age group provide an appropriate number of degrees of freedom for the planned statistical model.

### 2.2. Collection of Tissue Samples

Veterinary inspection carried out by the veterinarian responsible for pre- and post-mortem examination confirmed the absence of pathological changes within the gastrointestinal tract in all animals. No experimental procedures were performed on live animals. All tissue samples were collected post-mortem in a certified commercial slaughterhouse. Therefore, ethical approval from the Local Ethics Committee was not required under applicable national regulations.

The material for analysis was collected in a certified cattle slaughterhouse. The animals were subjected to an 18 h fast prior to slaughter. Immediately after slaughter, samples from the gastrointestinal tract were collected, including the abomasum, duodenum, jejunum, and ileum. Care was taken to ensure that each sample contained all layers of the gastrointestinal wall. Well-preserved fragments from each segment were collected to ensure representativeness of the material.

Samples intended for immunohistochemical analyses were immediately rinsed with physiological saline to remove impurities and then placed in 4% formaldehyde (pH 7) for 24 h. After fixation, samples were rinsed under running water and then dehydrated in a graded ethanol series. After the clearing step in xylene, tissues were infiltrated with paraffin and embedded using a modular embedding station (MYR EC-350, Casa Álvarez Material Científico SA, Madrid, Spain).

From the prepared paraffin blocks, 5 µm sections were cut using a rotary microtome (HM 360, Microm, Walldorf, Germany). Every fifth section was mounted on SuperFrost^®^ Plus slides (Thermo Scientific, Menzel-Glaser, Braunschweig, Germany) and placed in an incubator (CG Wamed, Warsaw, Poland) at 37 °C for 12 h.

### 2.3. Immunohistochemical Analysis (IHC)

Immunohistochemical staining was performed using rabbit polyclonal antibodies directed against specific antigens (listed in Table 1). The sections were deparaffinized in xylene, rehydrated in ethanol of decreasing concentrations (100–50%), and rinsed in deionized water. Endogenous peroxidase activity was then blocked with 3% hydrogen peroxide H_2_O_2_ (15 min at room temperature), and antigen retrieval was carried out in citrate buffer (pH 6.0) using a pressure cooker (8 min, 120 °C, multicooker RMC-PM381-E, Redmond Industrial Group, Moscow, Guangzhou, China). After an additional 5 min blocking of nonspecific binding in the UltraCruz Blocking Reagent (sc-516214, Santa Cruz Biotechnology, Dallas, TX, USA), the sections were incubated overnight at 4 °C with the primary antibody. The next day, a two-step detection system was applied using a secondary antibody conjugated with horseradish peroxidase (HRP) (ImmunoLogic WellMed BV, Duiven, The Netherlands), followed by visualization with 3,3′-diaminobenzidine (DAB substrate kit; ab64238; Abcam, Cambridge, UK). The slides were counterstained with Mayer’s hematoxylin (Patho, Mar-Four, Konstantynów Łódzki, Poland), dehydrated, cleared in xylene, and coverslipped. In the negative controls, in which the primary antibody was replaced with phosphate-buffered saline (PBS), no staining reaction was observed. The immunohistochemical staining protocol used in this study was based on the method described by Janicka et al. [29].

### 2.4. Histomorphometric Analysis

Microscopic analysis was performed on stained histological sections. Microscopic evaluations were conducted using a light microscope (BX-51 DSU, Olympus, Tokyo, Japan) equipped with a color digital camera (DP-70, Olympus, Tokyo, Japan) at magnifications of 100×, 200× and 400×. High-resolution digital images were captured using Cell^M 2.3 software (cellSens Standard, Olympus, Tokyo, Japan) under constant illumination and uniform brightness and contrast settings by a single operator. The images were analyzed using ImageJ software (version 1.54f, National Institutes of Health, Bethesda, MD, USA; http://rsb.info.nih.gov/ij/index.html, accessed 22 November 2023).

The study was conducted in two age groups of cattle: young and adult animals. For gastrin, SOM, and CgA in the abomasum, 20 randomly selected, well-preserved gastric glands were analyzed in each individual from both age groups. The glands were selected randomly, including only structures with properly preserved morphology. Only complete glandular profiles, defined as structures entirely contained within the field of view, were included in the analysis. To avoid distortion of the results resulting from differences in gland size, all immunoreactive cells visible in the plane of section were counted in each analyzed gland, and the surface area of each gland was measured. The results were expressed as the mean number of immunoreactive cells per standardized area of 50,000 µm^2^ of a gastric gland.

For GIP, GLP-1, secretin, SOM, and CgA in the small intestine, a grid-based quantitative method was applied. Non-overlapping fields were randomly selected within the intestinal mucosa. Only well-preserved areas without histological artifacts were included in the analysis. Measurement grid implemented in ImageJ software was used to define the analyzed area. In each field, 100 grid squares covering intact mucosa were evaluated to ensure a standardized assessment of the mucosal surface. A measurement grid consisting of 100 squares (each 5000 µm^2^) was superimposed on the mucosal surface, resulting in a total analyzed area of 0.5 mm^2^ per field. In each animal, 10 fields per intestinal segment were analyzed. All clearly identifiable immunoreactive cells within the defined area were counted, and results were expressed as the mean number of positive cells per analyzed mucosal area.

Different analytical approaches were applied due to structural differences between glandular and intestinal mucosa.

To minimize observer variability, all measurements were performed by the same person using constant and clearly defined evaluation criteria. Only mucosal regions with properly preserved architecture, sectioned perpendicular to the surface, were included in the morphometric analyses. Deformed, damaged, or improperly oriented fragments were excluded from further evaluation. Each measurement was performed by the same observer after prior standardization of section orientation to ensure proper alignment of structures relative to the section plane. For statistical analysis, the individual animal was treated as the experimental unit, and values obtained from multiple glands or microscopic fields were averaged per animal prior to inferential testing.

### 2.5. Statistical Analysis

All statistical analyses were performed using GraphPad Prism 10.5.0 for Windows (GraphPad Software, San Diego, CA, USA). Data are presented as mean ± standard error of the mean (SEM). For model-based analyses, results are reported as estimated marginal means with 95% confidence intervals. Statistical significance was set at *p* < 0.05. For the small intestine, the numbers of immunoreactive cells in the duodenum, jejunum, and ileum were analyzed using a two-way repeated-measures ANOVA in GraphPad Prism version 10.5.0 for Windows (GraphPad Software, San Diego, CA, USA), with age group (calves vs. adult bulls) as the between-subject factor and intestinal segment (duodenum, jejunum, ileum) as the within-subject repeated factor, including the age × segment interaction. Because all three intestinal segments were obtained from the same animal, the analysis was matched by animal, and the individual animal was treated as the experimental unit. Assumptions of normality and homogeneity of variances were assessed using the Shapiro–Wilk test and Levene’s test, respectively. When these assumptions were not met, data were subjected to logarithmic transformation, and the assumptions were reassessed on the transformed data. If a significant age × segment interaction was detected, main effects were not interpreted separately; instead, simple effects were examined within the same model, followed by Tukey-adjusted multiple comparisons. When the interaction was not significant, the main effects of age and intestinal segment were interpreted, and Tukey’s post hoc test was used for pairwise comparisons as appropriate. For the abomasum, where immunoreactive cells were quantified per 50,000 µm^2^, comparisons between calves and adult bulls were analyzed separately. When data met the assumptions of parametric testing, an unpaired Student’s *t*-test was applied. If the assumptions of normal distribution were not fulfilled, the Mann–Whitney U test was used as the nonparametric alternative.

## 3. Results

**Figure 1:**
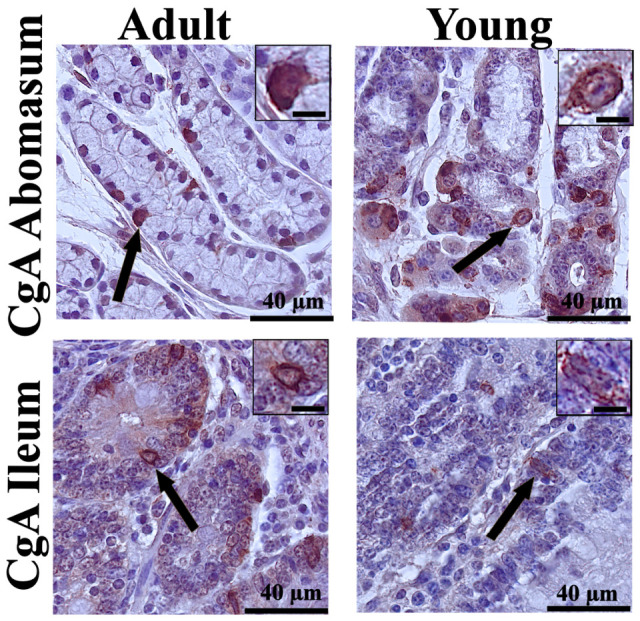
Representative photomicrographs of immunohistochemical reactions for chromogranin A (CgA) in the abomasal glands and in the ileal mucosa of young and adult bulls. Black arrows indicate chromogranin A-immunoreactive enteroendocrine cells. In the top-right corner, a magnified view of the reaction. Scale bar: 40 µm for the main image and 15 µm for the magnified reaction inset.

**Figure 2:**
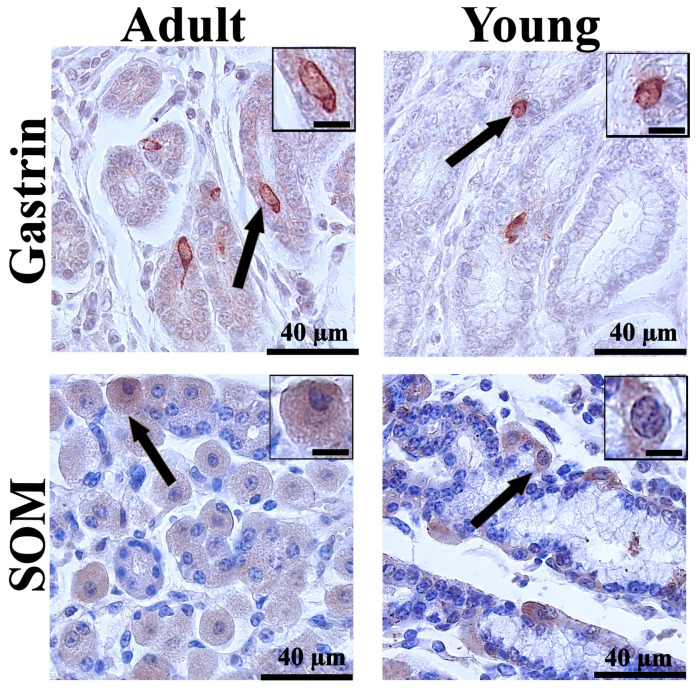
Representative photomicrographs of immunohistochemical reactions for gastrin and somatostatin (SOM) in the abomasum of young and adult bulls. Black arrows indicate immunoreactive enteroendocrine cells. In the top-right corner, a magnified view of the reaction. Scale bar: 40 µm for the main image and 15 µm for the magnified reaction inset.

**Figure 3:**
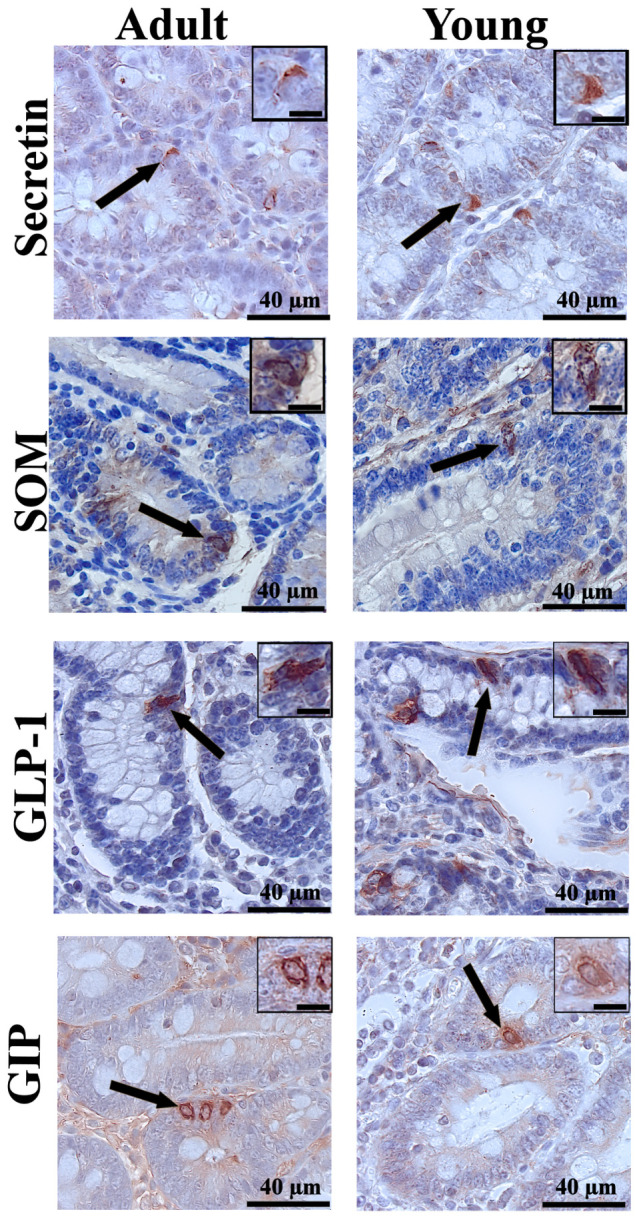
Representative photomicrographs of immunohistochemical reactions for secretin (SEC) in the duodenum, somatostatin (SOM) in the jejunum, glucagon-like peptide-1 (GLP-1) in the ileum, and glucose-dependent insulinotropic polypeptide (GIP) in the duodenum of young and adult bulls. Black arrows indicate immunoreactive enteroendocrine cells within the intestinal mucosa. In the top-right corner, a magnified view of the reaction. Scale bar: 40 µm for the main image and 15 µm for the magnified reaction inset.

The immunohistochemical analysis revealed differences in the distribution of enteroendocrine cells between young and adult cattle, as well as between gastrointestinal segments. CgA, used as a general marker of enteroendocrine cells, demonstrated their presence in all examined segments. Representative immunoreactivity in the abomasum and ileum is shown in Figure 1. In contrast, specific gastrointestinal hormones showed distinct distribution patterns in the abomasal glands (Figure 2) and in individual segments of the small intestine (Figure 3).

### 3.1. CgA in the Abomasum and Small Intestine

**Figure 4:**
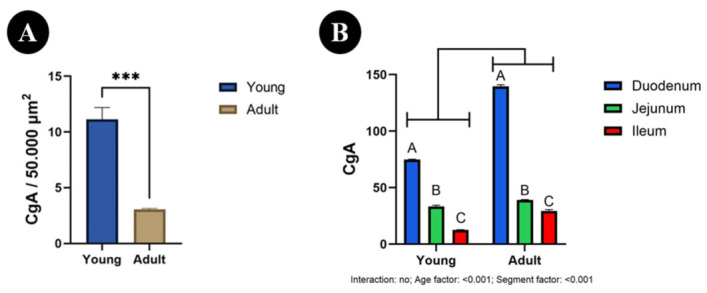
Number of chromogranin A (CgA) immunoreactive cells in the abomasum (**A**) and in the duodenum, jejunum, and ileum (**B**) of calves and adult bulls. The number of immunoreactive cells was expressed per 50,000 μm^2^ of glandular area in the abomasum and per 500,000 μm^2^ (0.5 mm^2^) of mucosal area in the intestine. Data are presented as mean ± SEM. Different letters (A–C) indicate statistically significant differences between intestinal segments within the same age group, whereas *** *p* < 0.001 indicates statistically significant differences between age groups.

In the abomasum, the number of CgA-immunoreactive cells was significantly higher in young animals [11.13; 95% CI] than in adults [3.04; 95% CI] (*p* < 0.001; Figure 4A).

In the small intestine, the number of CgA-immunoreactive cells showed no significant age × segment interaction (*p* = 0.143), but significant effects of age (*p* < 0.001) and segment (*p* < 0.001) were detected (Figure 4B). Overall, the number of CgA-immunoreactive cells was higher in adults than in young animals. In both age groups, the highest values were found in the duodenum [139.7 vs. 74.77; 95% CI], lower values in the jejunum [39.10 vs. 33.38; 95% CI], and the lowest in the ileum [29.37 vs. 12.53; 95% CI] (*p* < 0.001 for all).

### 3.2. Abomasum

**Figure 5:**
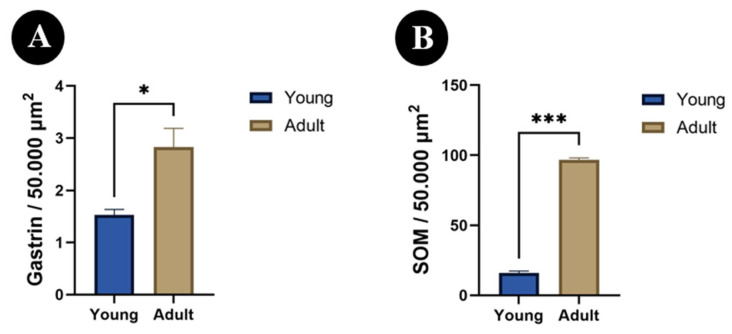
Number of gastrin (**A**) and somatostatin (SOM) (**B**) immunoreactive cells in the abomasum of calves and adult bulls. The number of immunoreactive cells was expressed per 50,000 μm^2^ of glandular area. Data are presented as mean ± SEM. * *p* < 0.05 and *** *p* < 0.001 indicate statistically significant differences between age groups.

The number of gastrin-immunoreactive cells within the analyzed area was significantly lower in young animals than in adults (*p* < 0.05; Figure 5A). A similar pattern was observed for SOM, as the number of SOM-immunoreactive cells was also significantly lower in young individuals than in adults (*p* < 0.001; Figure 5B).

### 3.3. Small Intestine

**Figure 6:**
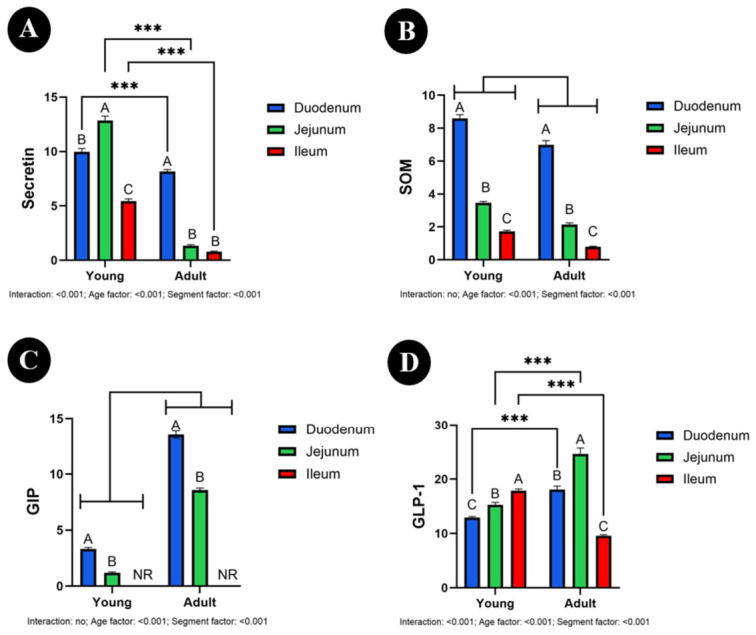
Number of secretin (**A**), somatostatin (SOM) (**B**), glucose-dependent insulinotropic polypeptide (GIP) (**C**), and glucagon-like peptide-1 (GLP-1) (**D**) immunoreactive cells in the duodenum, jejunum, and ileum of calves and adult bulls. The number of immunoreactive cells was expressed per 500,000 μm^2^ (0.5 mm^2^) of mucosal area. Data are presented as mean ± SEM. Different letters (A–C) indicate statistically significant differences between intestinal segments within the same age group, whereas *** *p* < 0.001 indicate statistically significant differences between age groups. NR—no reaction.

In the small intestine, the number of secretin-immunoreactive cells showed a significant age × intestinal segment interaction (*p* < 0.001; Figure 6A). Therefore, the effects were interpreted by examining simple comparisons within age groups and within individual intestinal segments. In young animals, the number of secretin-immunoreactive cells was highest in the jejunum, lower in the duodenum, and lowest in the ileum (*p* < 0.001 for all). In adult animals, the highest value was noted in the duodenum (*p* < 0.001), whereas the jejunum and ileum showed significantly lower numbers of immunoreactive cells (*p* = 0.260). Comparisons between age groups within individual intestinal segments showed that the number of secretin-immunoreactive cells was significantly higher in young than in adult animals in the duodenum [9.96 vs. 8.15; 95% CI], jejunum [12.85 vs. 1.35; 95% CI], and ileum [5.43 vs. 0.80; 95% CI] (all *p* < 0.001).

The number of SOM-immunoreactive cells showed no significant age × segment interaction (*p* = 0.130), whereas both the age factor (*p* < 0.001) and the segment factor (*p* < 0.001) were significant (Figure 6B). Overall, the number of SOM-immunoreactive cells was higher in young than in adult animals. In both age groups, the highest number of immunoreactive cells was observed in the duodenum [8.58 vs. 6.98; 95% CI], intermediate values in the jejunum [3.46 vs. 2.13; 95% CI], and the lowest values in the ileum [1.71 vs. 0.78; 95% CI] (*p* < 0.001 for all).

Similarly, the number of GIP-immunoreactive cells was not affected by the age × segment interaction (*p* = 0.072), whereas both the age factor (*p* < 0.001) and the segment factor (*p* < 0.001) were significant (Figure 6C). Overall, the number of GIP-immunoreactive cells was higher in adult [duodenum 13.53, jejunum 8.56; 95% CI] than in young [duodenum 3.31, jejunum 1.18; 95% CI] animals. In both age groups, the duodenum showed higher numbers of immunoreactive cells than the jejunum (*p* < 0.001 for all), whereas no immunoreaction was observed in the ileum.

In contrast, the number of GLP-1-immunoreactive cells showed a significant age × intestinal segment interaction (*p* < 0.001; Figure 6D). Therefore, the results were interpreted based on simple comparisons within age groups and within individual intestinal segments. In young animals, the number of GLP-1-immunoreactive cells was highest in the ileum (*p* < 0.001), intermediate in the jejunum (*p* = 0.010), and lowest in the duodenum (*p* = 0.021). In adults, the highest value was observed in the jejunum, followed by the duodenum, while the ileum showed the lowest number of immunoreactive cells (*p* < 0.001 for all). Comparisons between age groups within segments demonstrated that the number of GLP-1-immunoreactive cells was significantly higher in adult than in young animals in the duodenum [18.07 vs. 12.90; 95% CI] and jejunum [24.61 vs. 15.24; 95% CI] (both *p* < 0.001), whereas in the ileum it was significantly higher in young [17.87; 95% CI] than in adult [9.60; 95% CI] animals (*p* < 0.001).

## 4. Discussion

### 4.1. Age-Dependent Changes in the Enteroendocrine System

Our study comparing the number of immunoreactive cells for CgA, gastrin, SOM, secretin, GIP, and GLP-1 in the gastrointestinal tract of young (7–8-month-old) and adult (20–24-month-old) cattle revealed clear age-dependent differences in the organization of the enteroendocrine system. The maturation of the ruminant gastrointestinal tract is accompanied not only by structural growth but also by progressive functional specialization of endocrine regulatory mechanisms responsible for coordinating digestion, nutrient absorption, and metabolic homeostasis [30], as well as by the gradual reorganization of enteroendocrine cell populations and changes in their secretory activity profile [31]. Although the gastrointestinal tract of cattle at 7–8 months of age is already fully functional, animals at this stage remain in a period of intensive growth and high metabolic activity, which may generate different regulatory requirements compared with mature individuals [30,32,33]. Importantly, the present study provides comprehensive comparative data on multiple enteroendocrine cell populations across different gastrointestinal segments in both young and adult cattle. Previous studies have typically focused on individual hormones or single developmental stages, therefore, the simultaneous evaluation of several endocrine markers in animals at distinct stages of postnatal development represents an important extension of current knowledge regarding the functional maturation of the ruminant gastrointestinal system.

### 4.2. Hormone-Specific Patterns of Enteroendocrine Cell

The pattern of enteroendocrine cell distribution observed in the present study may reflect differences in the functional requirements of particular segments of the gastrointestinal tract associated with the stage of organismal development. Specifically, higher CgA immunoreactivity was observed in the small intestine of adult animals, whereas higher levels of this marker were detected in the abomasum of younger individuals. The abomasum plays a particularly important role in regulating proteolysis processes and controlling the digestive environment [34], which may explain the higher CgA immunoreactivity in this segment of the gastrointestinal tract in younger animals and reflect greater regulatory plasticity of this organ during the period of intensive growth [35,36]. As physiological maturity is reached and growth rate stabilizes, the importance of the small intestine gradually increases as a key site of hormonal regulation of digestion and metabolism. This is because the small intestine constitutes the main site of nutrient absorption and integration of hormonal signals influencing the functioning of the entire organism [37]. Consequently, a greater number of CgA-immunoreactive cells in the small intestine of adult cattle may reflect a higher degree of functional specialization of this segment of the gastrointestinal tract. It may also indicate differences in the organization of enteroendocrine cell populations in this region. However, CgA represents only a general marker of enteroendocrine cells, and the distribution of specific hormone-immunoreactive cell populations observed in the present study indicates variability dependent on both intestinal segment and animal age [38]. This result also suggests that the endocrine system of the gastrointestinal tract continues to develop even after the completion of early growth stages, adapting to the increasing complexity of digestive and metabolic processes, since intestinal maturation is accompanied by changes in cell populations and regulatory functions controlled by hormonal and nutritional factors [39]. The results of our study (the highest number of CgA-immunoreactive cells in the duodenum, with their number gradually decreasing toward the ileum in both young and adult individuals) are consistent with observations reported by other authors. Similar findings were obtained by both Alumare [40] and Swadi and Rammah [41], who demonstrated the presence of CgA-immunoreactive cells in the mucosa of the small intestine of ruminants along its entire length, with the greatest number of cells in the proximal segments of the intestine, particularly in the duodenum and jejunum, and a smaller number in distal segments. In the study by Alumare [40], CgA-positive cells were localized mainly in the duodenum and jejunum, whereas their number was clearly lower in the ileum. In turn, Swadi and Rammah [41] demonstrated the presence of CgA-immunoreactive cells in the proximal, middle, and distal parts of the small intestine of the camel, with their distribution indicating a numerical predominance in proximal segments. While previous studies described the regional distribution of CgA-immunoreactive cells in ruminants, the present study extends these observations by demonstrating clear age-related differences in the localization of these cells between the abomasum and small intestine. These findings highlight the dynamic reorganization of the enteroendocrine system during development and provide quantitative evidence of functional specialization of individual gastrointestinal segments with increasing age.

One of the most pronounced findings of the present study was significantly higher immunoreactivity of gastrin and SOM in the abomasum of adult cattle compared with younger individuals, while maintaining a clear decreasing gradient from the duodenum to the ileum in both age groups. Such a distribution pattern of cells in adult animals suggests the establishment of a more stable and precisely controlled digestive environment characteristic of the fully developed physiology of the ruminant gastrointestinal tract. In the mature organism, the abomasum plays a key role in the initial stage of protein digestion following microbial fermentation occurring in the forestomachs, which requires effective regulation of acid and proteolytic enzyme secretion [34]. Moreover, Guilloteau et al. [3] reported that hydrochloric acid (HCl) secretion in the abomasum is low around birth and increases during the first days of life, accompanied by changes in the pH of abomasal contents. Since gastrin is a classical stimulator of hydrochloric acid secretion, the lower number of gastrin cells observed in our study in younger animals may be associated with the maturation of the secretory function of the abomasum Available comparative quantitative data on gastrin- and SOM-immunoreactive cells in young and adult cattle across multiple gastrointestinal segments remain scarce. Therefore, the present findings provide new evidence supporting the role of age-dependent endocrine regulation in the maturation of the ruminant digestive system. The pattern observed in the present study, consisting of higher CgA immunoreactivity in the abomasum of younger animals together with lower gastrin and SOM immunoreactivity compared with adult individuals, may result from differences in the nature of digestive regulation associated with the stage of physiological development of the organism rather than from immaturity of the gastrointestinal tract [17,42]. Under such conditions, an increased number of enteroendocrine cells in the abomasum may reflect a greater need for rapid regulatory adaptation. This may help maintain adequate efficiency of digestive processes during periods of intensified anabolic activity [1]. In adult individuals, mechanisms stabilizing the digestive environment and maintaining metabolic balance under conditions of stabilized growth rate and more predictable feed intake play a greater role [43]. In the small intestine, the number of SOM cells was generally higher in younger animals, and their distribution showed a consistent proximal-to-distal gradient (duodenum–jejunum–ileum) in both age groups. This pattern is consistent with the role of SOM as a local regulator inhibiting secretory and motor processes of the gastrointestinal tract [18]. Higher levels of this hormone in younger animals may reflect the need for more intensive control of digestive processes during periods of rapid growth and greater variability in metabolic conditions [44]. At the same time, studies by Lucini et al. [45] in water buffalo indicate that the number of SOM cells in the intestine may remain relatively stable with age, suggesting the possibility of interspecies differences or dependence of observed changes on the developmental stage of animals. In contrast to reports describing relatively stable SOM cell numbers in some ruminant species, the present results indicate a clear age-related difference in the small intestine of cattle, suggesting species-specific patterns of endocrine adaptation and highlighting the importance of developmental stage as a determinant of intestinal regulatory mechanisms.

Secretin immunoreactivity in the small intestine was higher in young animals than in adults in all analyzed intestinal segments. Secretin plays an important role in regulating bicarbonate secretion by the pancreas and maintaining an appropriate pH in the intestinal lumen [12]. The increased number of immunoreactive cells for this hormone in younger animals may indicate greater sensitivity of the developing gastrointestinal system to changes in the acidity of intestinal contents and the need for more intensive regulation of digestive processes during growth. In the young organism, mechanisms controlling the intestinal environment are particularly important because the gastrointestinal tract undergoes adaptation to a diet of increasing complexity [46]. Our data regarding the predominance of secretin cells in younger animals are also consistent with observations by Guilloteau et al. [3], who demonstrated that during early life the pH environment in the gastrointestinal tract undergoes intensive changes. The authors reported that in newborns the pH of intestinal contents ranges from approximately 5.5 to 6.5, whereas in older calves, these values remain mainly in the proximal duodenum, while in the jejunum and ileum, they reach approximately 7.0 to 8.0. However, it should be taken into account that enteroendocrine cells may exhibit co-expression of multiple hormones. Hysenaj et al. [47] indicate that secretin expression may be an element of enteroendocrine cell maturation, and the number of immunoreactive cells may reflect changes in hormonal expression rather than solely changes in cell number. Our results complement these observations by providing evidence of increased secretin-immunoreactive cell density in young animals, supporting the concept of enhanced endocrine responsiveness of the developing gastrointestinal tract during periods of rapid growth.

The number of GIP-immunoreactive cells was greater in adult animals than in young ones, and in both age groups the highest number of cells was observed in the duodenum, fewer in the jejunum, whereas no GIP-immunoreactive cells were found in the ileum. This result suggests that with age there is a strengthening of mechanisms regulating nutrient utilization and control of energy metabolism [14]. Our findings confirm previous observations regarding the proximal predominance of GIP-immunoreactive cells. However, the present study additionally indicates an age-dependent increase in these cells, indicating progressive maturation of metabolic regulatory mechanisms in adult cattle and providing quantitative support for the functional transition from growth-oriented metabolism to stabilized energy homeostasis. GIP plays a key role in stimulating insulin secretion and regulating anabolic processes. Therefore, the greater number of immunoreactive cells in adult animals may reflect a transition from growth-oriented metabolism to more stable regulation of energy homeostasis [48]. Our findings confirm observations of other authors indicating that GIP-immunoreactive cells occur most abundantly in the duodenum, their number decreases markedly in the jejunum, whereas in the ileum they are few or absent [16,40,49]. In contrast to GIP, GLP-1 immunoreactivity showed a complex pattern dependent on both age and intestinal segment. In young animals, the highest immunoreactivity was observed in the ileum, whereas in adults, it was observed in the jejunum. Such a shift may reflect the development of nutrient detection mechanisms and changes in intestinal transit dynamics during maturation of the gastrointestinal tract [50]. The observed shift in the predominant localization of GLP-1-immunoreactive cells from distal intestinal segments in young animals to more proximal segments in adult cattle suggests a previously underappreciated age-related shift in this species and may reflect functional maturation of nutrient sensing and hormonal regulation during development. In addition to developmental maturation, the observed differences may also reflect the high functional plasticity of the ruminant gastrointestinal tract [30]. According to Elsabagh and Sugino [51], the enteroendocrine system responds dynamically to changes in diet composition, microbial activity, and metabolic demands of the organism. With age, rumen function develops and the microbiota stabilizes, leading to more complex hormonal regulation of digestive processes [52]. In a study conducted in sheep by Awadha and Hassan [53], it was demonstrated that the number of GLP-1-immunoreactive cells increased toward the distal segments of the small intestine and was highest in the ileum, whereas it was lowest in the duodenum. These findings are consistent with observations obtained in the present study in young animals, in which the highest number of GLP-1 cells was also found in the ileum. In an ontogenetic study of the bovine gastrointestinal tract, Pyarokhil et al. [54] demonstrated a different regional pattern in which GLP-1-immunoreactive cells were more numerous in the duodenum and jejunum, and their number decreased in the ileum. The authors also reported that the number of GLP-1 cells was highest in fetuses and then decreased in calves and adult animals, indicating significant changes in the distribution of these cells during postnatal development. This pattern is consistent with the results obtained in the present study in adult animals, in which the highest number of GLP-1 cells was also observed in the jejunum and the lowest in the ileum. Altogether, the results of our study and the literature data indicate that the distribution of GLP-1-immunoreactive cells in the small intestine changes during postnatal development. In young animals, these cells are relatively more prevalent in distal segments, whereas in adult individuals they are more common in proximal segments. Such changes may reflect functional maturation of the gastrointestinal tract and adaptation to changing feeding patterns and metabolic demands at successive stages of life [54].

### 4.3. Functional Significance of Enteroendocrine Maturation

The results indicate that maturation of the gastrointestinal tract is accompanied by coordinated changes in the number of enteroendocrine cells, reflecting the gradual functional specialization of the digestive system and its adaptation to changing metabolic demands. The present study provides comprehensive data on the age- and region-specific distribution of multiple enteroendocrine cell populations in cattle, offering a valuable reference for future experimental and comparative studies investigating digestive physiology, endocrine regulation, and nutritional management in ruminants.

### 4.4. Limitations of the Study

It is worth noting that the present study had a cross-sectional design and included a relatively small number of animals (six individuals in each group). All analyzed animals were male Holstein–Friesian cattle originating from a single farm and maintained within the same production system. The selection of males only allowed for the reduction in the potential influence of hormonal factors associated with the estrous cycle in females, which may affect the functioning of the gastrointestinal tract and the activity of endocrine cells. Therefore, the obtained results should be interpreted with caution, and their generalization to other cattle populations, different breeds, production systems, or environmental conditions may be limited. At the same time, the study provides preliminary data on the immunoreactivity of enteroendocrine cells in the abomasum and small intestine of cattle maintained under typical production conditions, which may serve as a basis for further studies involving larger groups of animals and more diverse populations.

It should be emphasized that the animals used in the study originated from commercial production farms and were intended for slaughter for consumption purposes rather than for experimental research. The cattle were maintained in a semi-intensive system, including a period of pasture grazing followed by feeding with a compound feed mixture. However, detailed data regarding the exact composition of the feed mixture, nutrient levels, and environmental parameters are not available. Therefore, the potential influence of nutritional factors on the analyzed parameters cannot be completely excluded. At the same time, the aim of the study was to present the characteristics of these parameters in cattle representing a population of animals commonly directed to slaughter under standard production conditions, rather than to evaluate the effect of a specific feeding model. The results indicate that the discussed approach may provide practical benefits for agriculture, which constitutes a basis for extending research to other livestock species, particularly cattle.

## 5. Conclusions

Clear differences in the number of immunoreactive enteroendocrine cells were observed between gastrointestinal segments and between age groups, indicating a region-specific and age-dependent distribution pattern. These findings expand current knowledge on the organization of enteroendocrine cells in ruminants and provide baseline data for further research. Importantly, the present study provides an integrated comparative analysis of multiple enteroendocrine cell populations across gastrointestinal segments in both young and adult cattle. The results highlight developmental stage and anatomical location as key determinants of endocrine organization in the bovine gastrointestinal tract and provide a foundation for future investigations into age-related physiological adaptation and optimization of feeding strategies in ruminant production systems.

## Figures and Tables

**Table 1:** Characteristics of antibodies used for immunohistochemical staining.

Target Antigen	Antibody Type	Host Species	Manufacturer (Country)	Catalog Number	Dilution
Anti-mouse/rabbit (secondary)	Polyclonal, HRP- conjugated, secondary	Goat	ImmunoLogic (Netherlands)	DPVB-HRP	RTU ^1^
GLP1 (Glucagon-like peptide-1)	Polyclonal, primary	Rabbit	Enzo (USA)	BML-GA1176-0100	1:500
GIP (Glucose-dependent insulinotropic polypeptide)	Polyclonal, primary	Rabbit	Invitrogen (USA)	PA5-76867	1:500
Secretin	Polyclonal, primary	Rabbit	USBiological (USA)	S0625-05K	1:500
Gastrin	Polyclonal, primary	Rabbit	Invitrogen (USA)	PA5-32422	1:200
SOM (Somatostatin)	Polyclonal, primary	Rabbit	Biorbyt (UK)	orb214613	1:900
CgA (Chromogranin A)	Polyclonal, primary	Rabbit	Abcam (UK)	ab85554	1:500

^1^ RTU—ready to use.

## Data Availability

All data are available within the manuscript.

## Abbreviations

The following abbreviations are used in this manuscript:

ANOVA	Analysis of Variance
CgA	Chromogranin A
DAB	3,3′-Diaminobenzidine
EECs	Enteroendocrine Cells
GLP-1	Glucagon-Like Peptide-1
GIP	Glucose-Dependent Insulinotropic Polypeptide
HCl	Hydrochloric acid
HRP	Horseradish Peroxidase
IHC	Immunohistochemistry
NR	No Reaction
PBS	Phosphate-Buffered Saline
RTU	Ready To Use
SEM	Standard Error of the Mean
SOM	Somatostatin
TMR	Total Mixed Ration
